# Paying in Blood: A Case of Thrombocytopenia in Covid-19

**DOI:** 10.7759/cureus.9791

**Published:** 2020-08-16

**Authors:** Jahanzeb Malik, Muhammad Javaid, Omaid Majedi, Uzma Ishaq, Tayyaba Zahid

**Affiliations:** 1 Cardiology, Rawalpindi Institute of Cardiology, Rawalpindi, PAK; 2 Hematology and Medical Oncology, Fauji Foundation Hospital, Rawalpindi, PAK

**Keywords:** covid 19, thrombocytopenia, covid associated thrombocytopenia

## Abstract

Novel coronavirus disease 2019 (COVID-19) primarily involves the respiratory system. Consumptive coagulopathy is seen in acute respiratory distress syndrome (ARDS) and multi-organ failure (MOF). Usually, only severely sick patients develop mild thrombocytopenia. We present a case of mildly symptomatic young adult, presenting with severe thrombocytopenia. She responded well to corticosteroids.

## Introduction

Since its advent in December 2019, the novel Coronavirus Disease 2019 (COVID-19) has infected thousands of people globally with nearly 5500 deaths [1]. COVID-19 primarily produces a respiratory and systemic illness progressing to acute respiratory distress (ARDS) and multi-organ failure (MOF) [2]. It can also involve other organ systems such as hematopoietic and the immune system [3]. 

Infection with COVID-19 typically presents with fever and upper respiratory symptoms like cough and dyspnea. A small proportion of mildly symptomatic patients present with atypical symptoms like diarrhea, thrombocytopenia, or bleeding tendencies [4]. Commonly, intravascular consumptive coagulopathies are only observed in severely ill patients [5]. A narrow threshold for the identification of COVID-19 should be practiced in the mild disease to prevent delayed diagnosis.

We present a case of COVID-19, diagnosed with an isolated thrombocytopenia.

## Case presentation

A 29-year-old previously healthy laboratory technician at our institute developed malaise and severe body aches for the past one day. There were no respiratory symptoms, fever, rigors, and chills. She did not report any loss of smell or taste and there were no gastrointestinal symptoms. She gave no history of substance abuse and did not take any medications. There was no history of bleeding or purpura. Her physical examination was unremarkable. 

**Figure 1:**
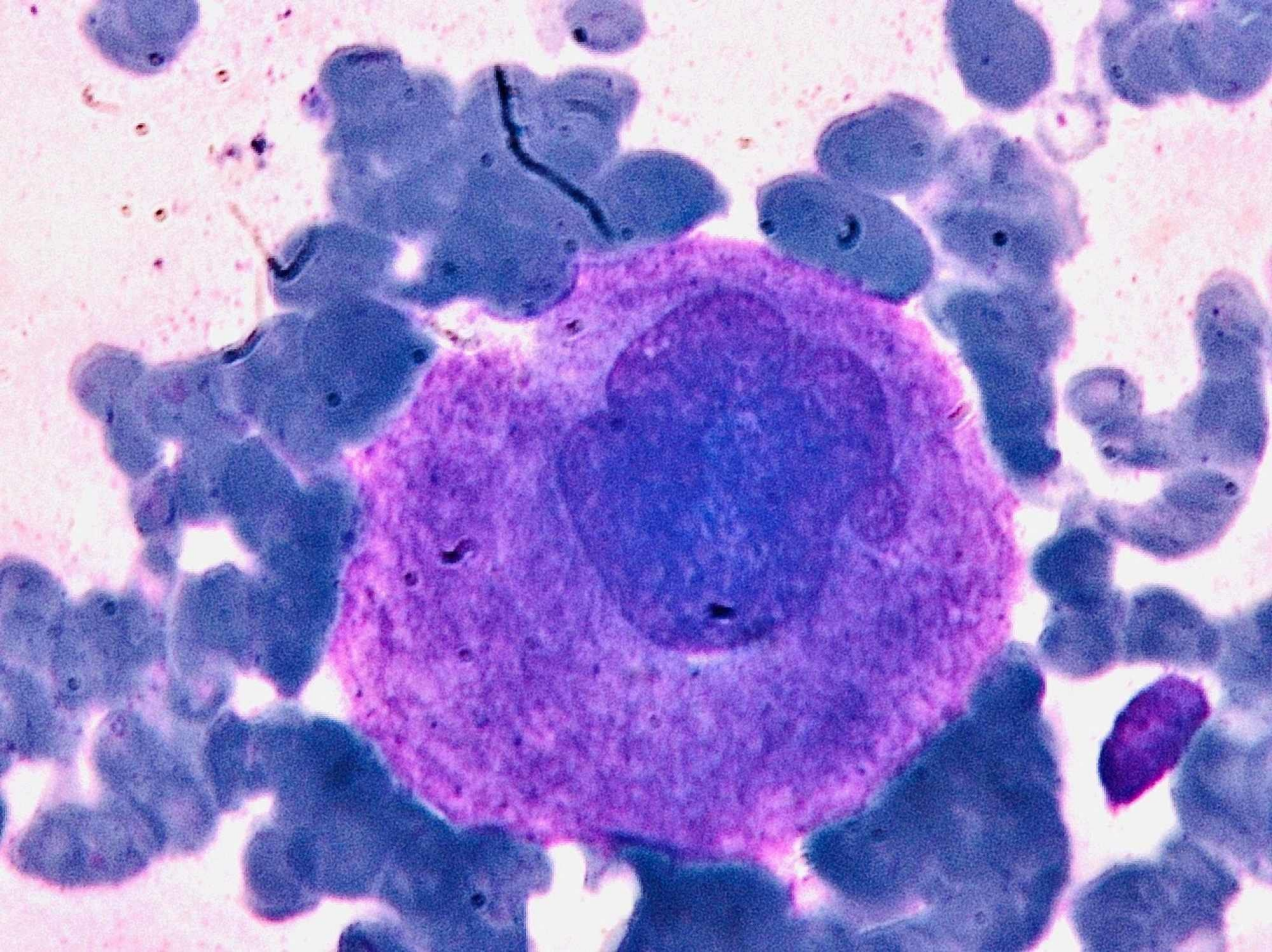
Megakaryocyte was identified on bone marrow aspirate at 100x power

**Figure 2:**
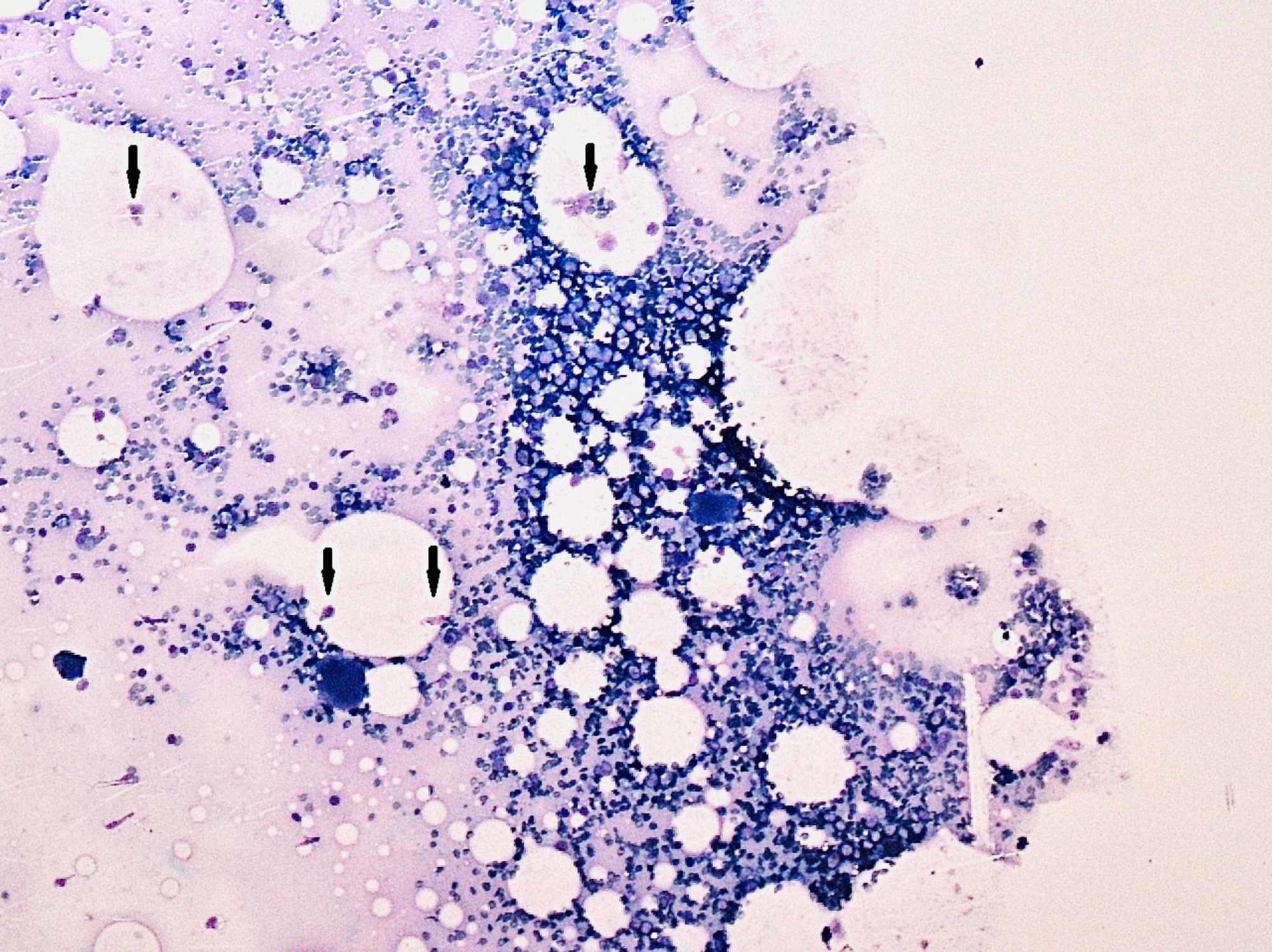
Multiple megakaryocytes are seen (Black arrows) on bone marrow aspirate at 10x power

**Figure 3:**
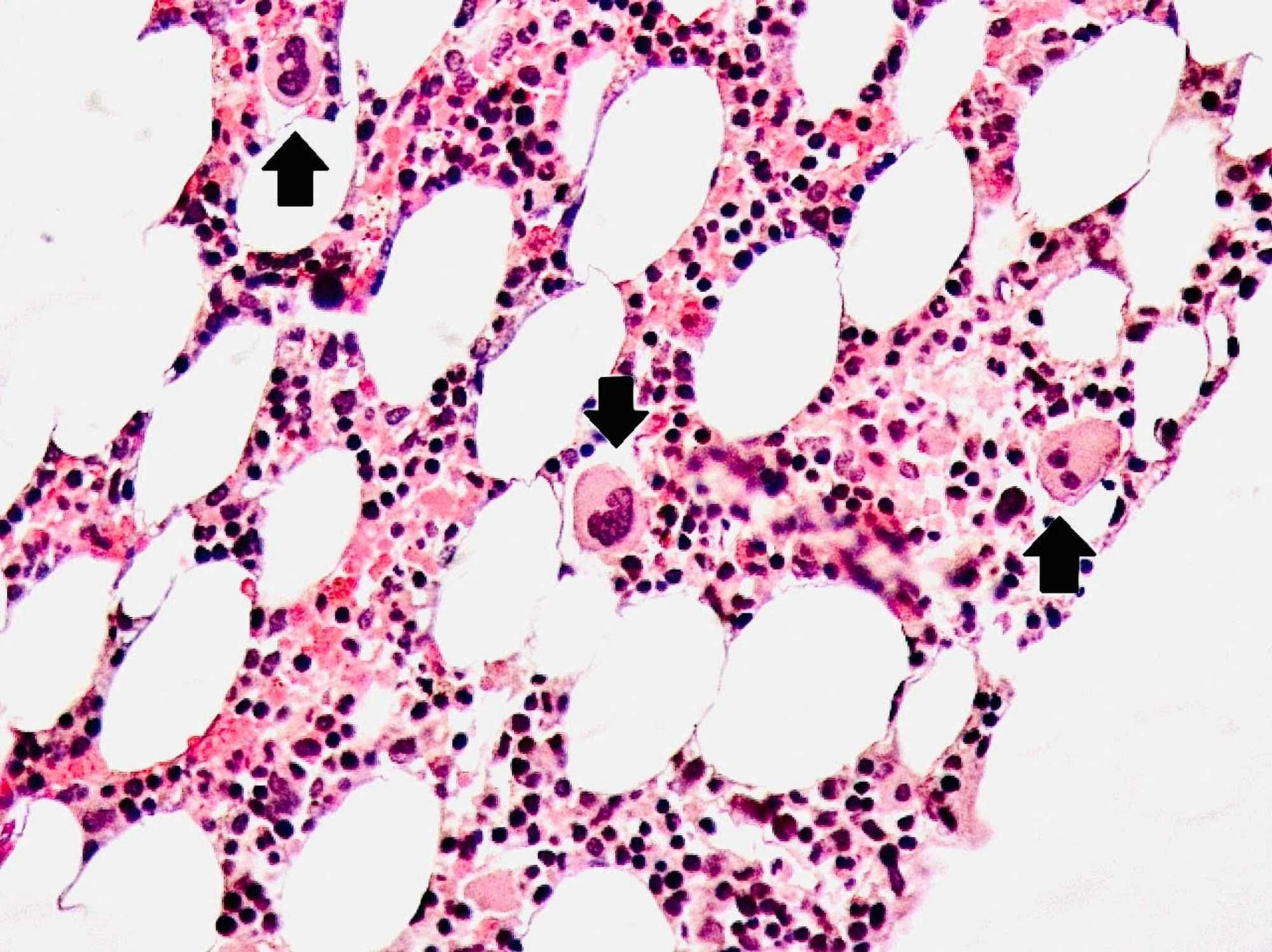
Megakaryocytosis confirmed on bone marrow trephine biopsy. This slide shows numerous megakaryocytes (Black arrows).

Hematology and biochemistry were ordered. The results showed significant thrombocytopenia. The platelet count was 20×10^9^/L and hemoglobin was 12.5 g/dl. There was a relative decrease in lymphocytes (12%). Her erythrocyte sedimentation rate (ESR) and C-Reactive Protein were 10 mm/hr and 6 mg/L while her D-dimers were negative. Peripheral blood smear showed decreased platelets of 18×10^9^/L and no schistocytes. Viral serology was negative for HCV-Ab, HIV-Ab, HBsAg and IgM, Herpes, and Cytomegalovirus. Abdominal ultrasound did not show visceromegaly. Chest X-ray and echocardiography were normal. NS-1 for dengue and a thick and a thin smear for malaria was also negative. A hematologist consult was made and a bone marrow biopsy showed increased megakaryocytes (Figures 1, 2, 3).

Considering the looming pandemic, her RT-PCR for COVID-19 was also sent, which came out to be positive. On the following day, her platelets dropped to 7×10^9^/L and there was myeloid predominance on the peripheral smear. She was transfused four units of platelets. Subsequent platelet counts improved to 12×10^9^/L. She was started on prednisolone 1 mg/kg in three divided doses. 

On the third day, her platelet count was stable at 12×10^9^/L. It increased to 16×10^9^/L on the fourth day and to 24×10^9^/L on the fifth day. On the seventh day, prednisolone was stopped as the platelet count reached 47×10^9^/L. Her second RT-PCR for COVID-19 was negative. She was discharged from our institute and on the first follow up after one week, her platelets were 166×10^9^/L. She remained stable on the subsequent follow-ups.

## Discussion

The exact mechanism of hematopoietic abnormalities is not known in COVID-19, but the proposed hypothesis is the increased autoantibodies and immune complexes clear the platelets from the circulation by the immune system. This leads to more platelet destruction and thrombocytopenia [6]. 

Several studies and case series have described a cascade of consumptive coagulopathies that cause thrombocytopenia [7, 8]. Such studies state that decreasing trend of platelets confer a grave prognosis of COVID-19 infection and lead to a severe disease [8]. One study has postulated that the virus significantly impacts the hematopoietic system leading to pancytopenia [9]. The observation in our patient could be an immunologic reaction commonly seen in viral infections. Surprisingly, all major studies have shown mild thrombocytopenia is a complicated course for the patient [10]. One study demonstrated severe thrombocytopenia with pulmonary and neurological complications. 

However, in mildly symptomatic patients, isolated thrombocytopenia is not frequently described. There is only one case report published recently on idiopathic COVID-19 associated thrombocytopenia [11]. Our patient was also mildly symptomatic and after ruling out all major causes of decreased platelets, it was postulated to be associated with the virus. 

## Conclusions

Variable presentations can lead to a missed diagnosis of COVID-19. This case report was intended to present a rare case and highlight the proposed mechanism for thrombocytopenia with the immune system working against the hematopoietic cell lines. The role of corticosteroids is evident in this disease and large randomized controlled trials should be conducted for better evidence as a treatment option. 
